# Optic nerve sheath fenestration for salvaging acutely threatened vision in Idiopathic Intracranial Hypertension - A two-year completed follow up

**DOI:** 10.12669/pjms.39.6.6441

**Published:** 2023

**Authors:** Sidrah Latif, Amna Rizwan, Asad Aslam Khan, Summan Zahra

**Affiliations:** 1 Sidrah Latif, FCPS Senior Registrar Ophthalmology Eye Unit-3, Institute of Ophthalmology, Mayo Hospital Lahore; 2 Amna Rizwan, FCPS Ophthalmology Eye Unit-3, Institute of Ophthalmology, Mayo Hospital Lahore; 3 Asad Aslam Khan, MS Ophthalmology Eye Unit-3, Institute of Ophthalmology, Mayo Hospital Lahore; 4 Summan Zahra, M.B.B.S Fourth-year student King Edward Medical University. Eye Unit-3, Institute of Ophthalmology, Mayo Hospital Lahore

**Keywords:** Idiopathic Intracranial Hypertension, Pseudotumor Cerebri, Optic nerve sheath fenestration, Optic Nerve Sheath Decompression

## Abstract

**Objectives:**

To determine the efficacy and safety of Optic Nerve Sheath Fenestration (ONSF) for salvaging acutely threatened vision in patients of Idiopathic Intracranial Hypertension (IIH).

**Methods:**

This retrospective, interventional case series study was performed at Institute of Ophthalmology Mayo Hospital Lahore from September 2017 to September 2019. Nine patients diagnosed with Idiopathic Intracranial Hypertension as per Modified Dandy Criteria, underwent medial trans-conjunctival ONSF. Pre-operative and 1^st^ day, 1^st^ week, 1^st^ month, 1^st^ year, and 2^nd^ year postoperative best-corrected logMAR visual acuities (BCVA) were recorded. Mean BCVA were calculated and compared using paired t-test. P-value <0.05 was taken as significant.

**Results:**

All nine patients were female with a mean age of 24 years. The average best-corrected pre-operative logMAR visual acuity (BCVA) in the better eye was 0.5 ± 0.28 and in the worse eye was 1.0 ± 0.57. After the worse eye ONSF, at 1^st^ week mean BCVA in better eyes was 0.27 ± 0.32 (p-value=0.001), while it was 0.43 ± 0.63 (p-value=0.006) in the worse eyes. At 2nd year follow-up after optic nerve sheath fenestration mean BCVA in better eyes was 0.30 ± 0.30 (p-value=0.002) and in worse eyes was 0.44 ± 0.63 (p-value=0.007). Four patients (44.4%) had a subconjunctival hemorrhage, two patients (22.2%) had binocular diplopia, one patient (11.1%) pre-septal cellulitis, and one patient (11.1%) had no improvement in vision because of pre-operative secondary optic atrophy. All patients had unilateral fenestration and bilateral improvement, six patients (66.67%) reported improvement in headache and successful tapering of medical therapy.

**Conclusion:**

Optic nerve sheath fenestration is effective as well as a safe surgical procedure to salvage acutely threatened vision in patients of Idiopathic Intracranial Hypertension on maximal medical treatment.

## INTRODUCTION

Idiopathic Intracranial Hypertension is defined as raised intracranial pressure in the absence of any identifiable cause.^1^ If a cause is found, it’s secondary intracranial hypertension. IIH is defined by the modified Dandy Criteria which was adopted from the work of Dandy in 1937.^2^ Subsequently, Friedman and Jacobson in 2002 and Friedman, Liu, and Digre in 2013 suggested altering the name of IIH to pseudo-tumor cerebri syndrome (PTCS)^3^, however, the alterations have been actively questioned from most quarters.^4^

For all practical purposes, the criteria laid down by Dandy; as signs and symptoms of raised intracranial pressure in an otherwise awake and alert patient, in the absence of any neurological signs (bilateral sixth nerve paresis being an exception), in the absence of any anatomical or physiological abnormalities of the CSF formation, circulation, drainage, and ventricular system, with normal magnetic resonance imaging and venography except for signs of raised ICP (empty Sella, stenosis of the transverse sinus, dilated optic nerve sheath and flattening of the posterior pole of the globe) and opening pressure of more than 250 mm Hg on Lumbar Puncture; is still used for diagnosing IIH.^5^

Symptoms of IIH range from positional headaches and tinnitus to transient visual obscurations associated with postural change.^6^ Papilledema is invariably discovered on examination. A false localizing sign (sixth nerve paresis) may be seen.^7^ The management involves a three-pronged approach; to treat any underlying disease, to reduce headache morbidity, and to preserve vision.^8^ While ventriculoperitoneal shunting is adopted for refractory headache, a surgical procedure of increasing importance to reduce progressive visual loss with maximal medical therapy and treat acutely threatened vision is optic nerve sheath fenestration.^9^

The mechanisms, by which an optic nerve sheath fenestration salvages vision, are variable. Unilateral fenestration reduces bilateral disc swelling and visual symptoms hence need for bilateral fenestration is redundant.^10^ However, optic nerve sheath fenestration is not effective for refractory headaches as the subarachnoid space around the optic nerve is sequestered from the intracranial subarachnoid space as proven by a concentration gradient of beta-trace protein, a lipocalin-like prostaglandin D-synthase (L-PGDS) between the two SAS.^11^ For refractory headaches, shunting stays the procedure of choice.

Optic nerve sheath fenestration is a safe and effective procedure for addressing visual deterioration in IIH. The complications reported are transient mostly, ranging from diplopia and tonic pupil to corneal dellen, conjunctival filtering bleb, and abscess, to more severe orbital apex syndrome, traumatic optic neuropathy, and orbital hematoma with nerve palsies.^12^ Sometimes a repeat procedure is required.

## METHODS

This study was conducted at Eye Unit-3 Institute of Ophthalmology, Mayo Hospital Lahore from September 2017 to September 2019. It was a retrospective case series. Non-probability purposive sampling was done.

### Ethical Approval:

This study was approved by the Institutional Review Board King Edward Medical University/Mayo Hospital Lahore. Ref.378/RC/KEMU dated 09-03-2022.

### Inclusion & Exclusion Criteria:

Patients diagnosed as Idiopathic Intracranial Hypertension following the modified Dandy criteria^2^ (signs and symptoms of raised ICP in awake/alert patient, no neurological deficits other than 6^th^ nerve paresis, no abnormality on MRI/MRV, no anatomical or physiological disturbance of CSF formation and circulation, opening CSF pressure more than 250 mm Hg) with progressive visual deterioration and papilledema on fundoscopy on maximal medical therapy (oral acetazolamide 250 mg QID) admitted in eye unit-3 for optic nerve sheath fenestration were included in the study. Patients with increased intracranial pressure due to a known cause were excluded from the study.

### Data collection procedure:

After approval from Institutional Review Board the patients fulfilling inclusion and exclusion criteria referred from the Neurology department who underwent Optic Nerve Sheath Fenestration under General Anesthesia previously, were included in the study. The hospital patient information system and charts were used to collect data. All patients, whose surgery was performed by one surgeon with a uniform surgical procedure were identified. Surgery was done using a medial transconjunctival approach disinserting medial rectus muscle. 4/0 silk traction sutures were passed at 12 o clock and 6 o clock, 1 mm from the limbus. Peritomy was done with relaxing incisions given till fornix.

The medial rectus muscle was identified and secured with vicryl 6/0 and disinserted and retracted with the conjunctiva. The eyeball was rotated laterally, two microsponges were used to advance between the eyeball laterally and disinserted MR medially to reach the posteromedial inferior quadrant of the globe. The optic nerve was identified and an opening was made into the sheath 1 mm posterior to the posterior pole of the globe to avoid peri-optic blood vessels. Egress of CSF was seen as a sign of successful fenestration. The margins of the fenestration were tweaked and enlarged to prevent premature closure before fibrosis set in. The medial rectus muscle was attached to its insertion. Conjunctiva was closed.

Post-operative antibiotic drops and anti-biotic steroid combination ointment were advised and follow-up was done on 1^st^ postoperative day, 1^st^ post-operative week, and 1^st^ post-operative month, whereupon patients were discharged to neurology care for their oral medicine continuation or tapering as per their headache profile. Patients were called for an annual review for two subsequent years. Visual acuity, pupillary reflexes, and papilledema grade were recorded. The local examination was done, and any complications from the procedure were noted and managed accordingly. The followup data was available from followup opd prescription slips. Consent for data was taken from the Head of concerned unit and telephonically from patients.

### Data Analysis:

Data was entered and analyzed using SPSS version 23. Qualitative variables like Gender, Improvement in visual acuity, and complications were presented as frequency and percentage. Quantitative variables like age, logMAR visual acuity, and grade of papilledema were presented as mean with standard deviation. Pre-operative and 1^st^ day, 1^st^ week, 1^st^ month, 1^st^ year, and 2^nd^-year post-operative visual acuities recorded were compiled. Mean BCVA were calculated. Paired t-test was applied and p-value <0.05 was considered significant.

**Table-I:** Descriptive Statistics Of Age And Gender.

Gender	n (%)	Minimum (years)	Maximum (years)	Mean ± SD (years)
Female	9 (100%)	17	40	24 ± 7.09
Male	0 (0%)	-	-	-

## RESULTS

Nine patients were included in the study. All patients n=9 (100%) were female. The mean age of the patients was 24 years, minimum and maximum being 17 years and 40 years respectively.

The average best-corrected pre-operative logMAR visual acuity (BCVA) in the better eyes was 0.5 ± 0.28 and in the worse eyes was 1.0 ± 0.57. After the worse eye optic nerve sheath fenestration, the visual acuities (BCVA) on the first postoperative day were 0.4 ± 0.34 in the better eye and 0.6 ± 0.59 in the worse eye. The difference between pre and postoperative mean logMAR visual acuity was insignificant in the better eye (p-value 0.11) however significant in the worse eye (p-value 0.01). At 1^st^ week post-surgery, the mean BCVA in better eyes was 0.27 ± 0.32, while it was 0.43 ± 0.63 in the worse eyes.

The p-value came out significant for better eye at 1^st^-week post-operative (p-value=0.001), as well as for worse eye (p-value=0.006). The vision stabilization achieved at 1^st^ week was carried through till the 1^st^ post-operative month follow-up and the difference from pre-operative BCVA was significant. At one-year follow-up after optic nerve sheath fenestration mean BCVA in better eyes was 0.30 ± 0.30 (p-value=0.002) and in worse eyes was 0.44 ± 0.63 (p-value=0.007). At two years follow-up after surgery, the values of one-year follow-up were consistent.

No serious complications were reported in any of the cases. Four patients (44.4%) had a noticeable subconjunctival hemorrhage on 1^st^ postoperative day which resolved completely by 1^st^-month follow-up. Two patients (22.2%) complained of post-operative binocular diplopia which improved in both patients within the first week of surgery. One patient (11.1%) had pre-septal cellulitis which resolved with the addition of oral antibiotic and anti-inflammatory agents. One patient (11.1%) had no improvement in vision because of pre-operative secondary optic atrophy. All patients underwent unilateral fenestration and reported bilateral improvement. Six patients (66.67%) reported improvement in headache and successful tapering of medical therapy as well.

**Table-II:** Logmar visual acuities in the better eye.

	Mean BCVA pre-operative (better eye) 0.5 ± 0.28	p-value
Mean BCVA 1^st^ day post-op	0.4 ± 0.34	0.11
Mean BCVA 1^st^ week post-op	0.27 ± 0.32	0.001*
Mean BCVA 1^st^ month post-op	0.27 ± 0.32	0.001*
Mean BCVA 1^st^ year post-op	0.30 ± 0.30	0.002*
Mean BCVA 2^nd^ year post-op	0.30 ± 0.30	0.002*

* (p-value <0.05 significant).

**Table-III:** logMAR visual acuities in worse (operated) eye

	Mean BCVA worse eye pre-operative 1.0 ± 0.57	p-value
Mean BCVA 1^st^ day post-op	0.60 ± 0.59	0.01*
Mean BCVA 1^st^ week post-op	0.43 ± 0.63	0.006*
Mean BCVA 1^st^ month post-op	0.43 ± 0.63	0.006*
Mean BCVA 1^st^ year post-op	0.44 ± 0.63	0.007*
Mean BCVA 2^nd^ year post-op	0.44 ± 0.63	0.007*

* (p-value <0.05 significant).

## DISCUSSION

Idiopathic Intracranial Hypertension was previously known as Benign Intracranial Hypertension^13^ but the name was discarded from use because of the significant visual morbidity associated with it.^14^ While most symptoms^5^ can be controlled with weight loss and medical management with acetazolamide^6^, acutely threatened vision and progressive visual loss despite maximum medical therapy needs surgical intervention.^8^ Ventriculoperitoneal shunting or lumbo-peritoneal shunting procedures were associated with 0.5% mortality.^15^ Optic nerve sheath fenestration emerged as a procedure with no procedure-related mortality, a much smaller surgical time, a simpler procedure, and early patient rehabilitation.^16^

Moreover, unilateral fenestration had bilateral effects, as opposed to the previous belief that a bilateral procedure may be required. It is believed to be due to the increased egress of CSF from the site of fenestration hence reducing the pressure exerted at that point.^17^ It may also be due to increased optic nerve head perfusion as indicated by doppler recorded postoperative increase in blood flow in posterior ciliary arteries.^18^ Furthermore, in later stages, as the site of fenestration develops fibrosis it is theorized to have a CSF barring effect from around the optic nerve head.^19^ Whatever may be the exact mechanism, optic nerve sheath fenestration is purported to reduce papilledema and visual symptoms.

These newer findings have put optic nerve sheath fenestration at the helm of visual salvage in refractory symptoms especially when headaches are not a prominent feature. In our study, the functional success defined as significant improvement in BCVA was seen in operated as well as the contralateral non-operated eye by 1^st^ post-operative week. Mean BCVA at a completed follow-up of two years was 0.30 ± 0.30 (p-value=0.002) in better eyes and 0.44 ± 0.63 (p-value=0.007) in worse eyes. No lasting complications from the procedure were reported. Mild complications of subconjunctival hemorrhage (44.4%) and post-operative diplopia (22.2%) resolved in the initial days after surgery. Six patients (66.67%) reported adequate control of headaches and tapering of oral medical therapy after fenestration for vision.

In a 2017 literature review done by Kalyvas et al,^16^ 15 case studies including 12 case series and three case reports were reviewed for the efficacy and safety profile of a total of 341 patients who underwent optic nerve sheath fenestration. The review didn’t segregate complications as per the choice of procedure (medial transconjunctival, lid splitting, lateral approaches). Out of 70 complications reported most were transient and the most recurring ones^12^ were diplopia, tonic pupil, conjunctival filtering bleb, subconjunctival hemorrhage, and a few reported orbital hematomas, cellulitis, and apex inflammation. However, the combined anatomical success rate was 95%, visual improvement in 67%, and 15.9% with initial improvement had a later deterioration mandating second procedure.

In a 2017 study conducted by Yaqub MA, in Pakistani population, same as our population, they reported improvement of vision in 77.4% eyes, out of a total of 18 patients, 13 of whom had bilateral fenestration. They completed a followup of one year. They also found significant and negative correlation between duration of symptoms before presentation and improvement in BCVA. They reported tonic pupil in eight eyes however we didn’t see this complication in our patients probably due to unilateral approach. Their inclusion criteria was not limited to strictly patients of IIH and broadened patient base to include secondary causes of raise in intracranial pressure.^20^

While our study is in agreement with the previous studies marking efficacy and safety of the procedure, the differences recorded are due to limited case numbers and variable surgical procedures. Only medial transconjunctival approach was used with medial rectus disinsertion in our study. No patient required a second procedure as the patient with no visual rehabilitation had secondary optic atrophy. This case series including only Idiopathic Intracranial Hypertension, and not including secondary causes of intracranial hypertension, was first of a kind in the local indigenous population and the results are encouraging to say the least.

This retrospective case series is the only one in this population with such a long completed followup, showing the consistency of results obtained over long time especially in cases of unilateral fenestration for controlling bilateral disease.

### Limitations of the Study:

It includes retrospective nature of data collection and small sample size and no representation of male gender in the data possibly owing to small sample size.

## CONCLUSION

Optic nerve sheath fenestration is effective as well as a safe surgical procedure to salvage acutely threatened vision in patients of Idiopathic Intracranial Hypertension on maximal medical treatment. Unilateral fenestration has bilateral positive results which are sustained on a long followup.

### Authors Contribution:

**SL and AAK** conceived, designed and did statistical analysis & editing of manuscript.

**AR, SZ**, did data collection and manuscript writing.

**SL** takes the responsibility and is accountable for all aspects of the work in ensuring that questions related to the accuracy or integrity of any part of the work are appropriately investigated and resolved.
